# On the Utility of MIBG SPECT/CT in Evaluating Cardiac Sympathetic Dysfunction in Lewy Body Diseases

**DOI:** 10.1371/journal.pone.0152746

**Published:** 2016-04-07

**Authors:** Hayato Odagiri, Toru Baba, Yoshiyuki Nishio, Osamu Iizuka, Minoru Matsuda, Kentaro Inoue, Akio Kikuchi, Takafumi Hasegawa, Masashi Aoki, Atsushi Takeda, Yasuyuki Taki, Etsuro Mori

**Affiliations:** 1 Department of Behavioral Neurology and Cognitive Neuroscience, Tohoku University Graduate School of Medicine, Sendai, Japan; 2 Department of Diagnostic Radiology, Tohoku University Hospital, Sendai, Japan; 3 Department of Radiology and Nuclear Medicine, Institute of Development, Aging, and Cancer, Tohoku University, Sendai, Japan; 4 Department of Neurology, Tohoku University Graduate School of Medicine, Sendai, Japan; 5 Sendai Nishitaga National Hospital, Sendai, Japan; Philadelphia VA Medical Center, UNITED STATES

## Abstract

**Background:**

Abnormal cardiac uptake of ^123^I-metaiodobenzylguanidine (^123^I-MIBG) is a diagnostic marker of Lewy body diseases (LBDs), e.g., Parkinson’s disease (PD) and dementia with Lewy bodies (DLB). Planar imaging is generally used to assess cardiac sympathetic dysfunction in ^123^I-MIBG scintigraphy; however, its clinical utility requires further improvement. We hypothesized that the co-registration of single-photon emission tomography (SPECT) and computed tomography (CT) images would improve the diagnostic accuracy of ^123^I-MIBG cardiac scintigraphy for LBDs. This study sought to evaluate the effects of SPECT/CT imaging on ^123^I-MIBG cardiac scintigraphy for diagnosing LBDs.

**Methods:**

We retrospectively investigated data of 54 patients (consecutive 18 patients in each PD, DLB, and idiopathic normal pressure hydrocephalus [iNPH] groups) who underwent ^123^I-MIBG cardiac scintigraphy (planar and SPECT/CT) because of suspected LBDs at the Tohoku University hospital from June 2012 to June 2015. We compared the diagnostic accuracies of the conventional planar ^123^I-MIBG method and SPECT/CT methods (manual and semi-automatic).

**Results:**

In the conventional planar analysis, ^123^I-MIBG uptake decreased only in the DLB group compared with the iNPH group. In contrast, the SPECT/CT analysis revealed significantly lower ^123^I-MIBG uptake in both the PD and DLB groups compared with the iNPH group. Furthermore, a receiver operating characteristic analysis revealed that both the manual and semi-automatic SPECT/CT methods were superior to the conventional planar method in differentiating the 3 disorders.

**Conclusions:**

SPECT/CT ^123^I-MIBG cardiac scintigraphy can detect mild cardiac sympathetic dysfunction in LDBs. Our results suggest that the SPECT/CT technique improves diagnostic accuracy for LBDs.

## Introduction

Parkinson’s disease (PD) and dementia with Lewy bodies (DLB) are common causes of gait disturbance and dementia in the elderly. These 2 disorders are exist on the same spectrum of Lewy body diseases (LBDs) [1]. Recent studies have demonstrated that LBDs are often accompanied by cardiac sympathetic nerve degeneration [2, 3]. This pathological change is thought to be highly specific to LBDs among neurodegenerative diseases. Therefore, findings suggestive of cardiac sympathetic denervation may have diagnostic significance in clinical practice.

^123^I-metaiodobenzylguanidine (^123^I-MIBG) cardiac scintigraphy is now widely used to assess cardiac sympathetic neuronal dysfunction in LBDs [4, 5]. ^123^I-MIBG is a physiological analogue of noradrenaline, and abnormal cardiac uptake of ^123^I-MIBG indicates cardiac sympathetic dysfunction. Recent studies have demonstrated that cardiac ^123^I-MIBG scintigraphy results are abnormal specifically in PD relative to other neurodegenerative movement disorders [6]. Furthermore, patients with DLB often exhibit low uptake on ^123^I-MIBG scintigraphy, and this finding is included as a supportive feature of the current DLB diagnostic criteria [7]. Despite its importance, the clinical utility of ^123^I-MIBG scintigraphy for the early diagnosis of LBDs is still debated [8, 9].

In general, manually defined regions of interest (ROIs) on planar images are conventionally used to calculate myocardial uptake of ^123^I-MIBG (heart-to-mediastinum ratio, H/M ratio) [5]. However, problems with the anatomical localization of the heart and the mediastinum on planar images lead to decreased sensitivity and specificity of cardiac scintigraphy for identifying cardiac sympathetic dysfunction. Recent radiological studies have demonstrated that co-registration of single-photon emission tomography (SPECT) and computed tomography (CT) images improves ROI localization [10, 11]. Based this background, a SPECT/CT hybrid system may improve the accuracy of ^123^I-MIBG scintigraphy for assessing cardiac function and thus aid in LBD diagnosis. This study sought determine the diagnostic effects of ^123^I-MIBG cardiac SPECT/CT imaging compared with conventional scintigraphic evaluation for diagnosing LBDs.

## Materials and Methods

### Participants

We enrolled consecutive 18 patients in each PD, DLB, and idiopathic normal pressure hydrocephalus (iNPH) groups in this study. The patients underwent cardiac ^123^I-MIBG SPECT/CT as a part of a routine assessment of motor slowing and cognitive impairment at the Tohoku University hospital from June 2012 to June 2015. iNPH is a common cause of parkinsonian gait and cognitive dysfunction that mimic LBDs [12]. Therefore, we included iNPH patients as non-LBD control subjects in this study. To exclude cases with comorbidities, we included shunt-responsive iNPH patients who did not have symptoms highly suggestive of LBDs [13]. The demographic and clinical characteristics are shown in Table 1. The PD group was significantly younger and had longer disease duration than the DLB and iNPH groups. There were no significant differences in sex between the 3 disease groups.

**Table 1:** Demographics.

	PD	DLB	iNPH	*P* value
Number	18	18	18	
Sex (female/male)	10/8	5/13	8/10	0.23^a^
Age (years)	64.7 ± 12.7	77.6 ± 6.3	77.1 ± 6.2	**^b^
Disease duration (years)	5.2 ± 2.6	3.0 ± 1.7	2.8 ± 1.6	*^b^
Hoehn and Yahr scale	2.6 ± 0.5	2.6 ± 0.9	2.6 ± 0.9	1.00 ^b^

a: Chi square test

b: One-way analysis of variance (ANOVA).

*, p < 0.005

**, p < 0.0001.

PD, Parkinson's disease; DLB, Dementia with Lewy bodies; iNPH, idiopathic normal pressure hydrocephalus

This study was retrospective analysis of de-identified patient data, therefore explicit patient consent is not necessary. Patients are informed of the use of their data via a public information leaflet (http://www.med.tohoku.ac.jp/public/doc/2015-1-224.pdf), and they have the option to opt out of the data collection and subanalysis. This study was approved by the ethical committee of the Tohoku University Graduate School of Medicine (Approval number: 2015-1-224).

### Assessment and diagnosis

The patients underwent detailed neurological and neuropsychological examinations and brain MRI in addition to cardiac scintigraphy. All diagnoses were performed by expert neurologists according to the UKPD Brain Bank criteria for PD [14], the consensus criteria for DLB [7], and the Japanese criteria for iNPH [15]. Detailed inclusion criteria in the iNPH group were as follows: (1) age at disease onset over 60 years; (2) the presence of more than one of the triad (gait disturbance, dementia, and urinary incontinence); (3) ventricular enlargement (Evans Index > 0.3) accompanied by narrowing of the subarachnoid CSF spaces in the high convexity areas; (4) normal CSF pressure and content; (5) the presence of symptoms suggestive of another disease that could account for their principal complaints, such as repeated visual hallucinations and marked fluctuations; (6) no previous illness that could have caused ventricular dilation; and (7) positive results on a CSF tap test and shunt-placement surgery. All PD patients met the clinical diagnostic criteria, all DLB patients fulfilled the probable DLB criteria, and all iNPH patients fulfilled the definite iNPH criteria. To compare severity of motor dysfunction among these diseases, patients were retrospectively evaluated with the Hoehn and Yahr scale. Patients with any factors that could potentially affect cardiac uptake of ^123^I-MIBG, such as poorly controlled diabetes and recent use of antidepressants [16], were excluded from this study.

### ^123^I-MIBG cardiac scintigraphy and SPECT/CT

Cardiac planar images were acquired 25 minutes (early image) and 3 hours (delayed image) after an intravenous injection of 111 MBq of ^123^I-MIBG (FUJIFILM RI Pharma Co, Ltd., Tokyo, Japan) using a dual-head gamma camera equipped with a parallel-hole low-energy high-resolution collimator (Symbia-T; Siemens, Erlangen, Germany). Planar images were obtained in 256×256 matrixes, the enlargement ratio was 1.0 (pixel size: 2.4×2.4 mm), and the acquisition time was 180 seconds. The energy discrimination was centered on 159 keV with a 20% window. The cardiac SPECT/CT scans were acquired and followed by planar imaging. The SPECT images were obtained in 64×64 matrixes, the enlargement ratio was 1.45 (pixel size: 6.59×6.59 mm), the acquisition time was 30 seconds, and 6 angles and 30 views were used. The detector system was interfaced with a dedicated nuclear medicine computer (esoft; Siemens). The collection window was 159 keV±10%, and the sub-window was 143 keV±8% or 175 keV±8% (scattered collection window). Image processing was performed on an image-processing workstation (Syngo MI workplace, version VA60B; Siemens AG).

### Image Analysis

#### Conventional H/M ratios on planar images

On each anterior planar ^123^I-MIBG image, the cardiac ROI was selected, and a rectangular mediastinal ROI was manually placed in the upper mediastinum by a nuclear medicine physician. Next, the heart-to-mediastinum (H/M) ratio was calculated by dividing the count density of the cardiac ROI by that of the mediastinal ROI.

### Manual ROI measurement on SPECT/CT

**Fig 1:**
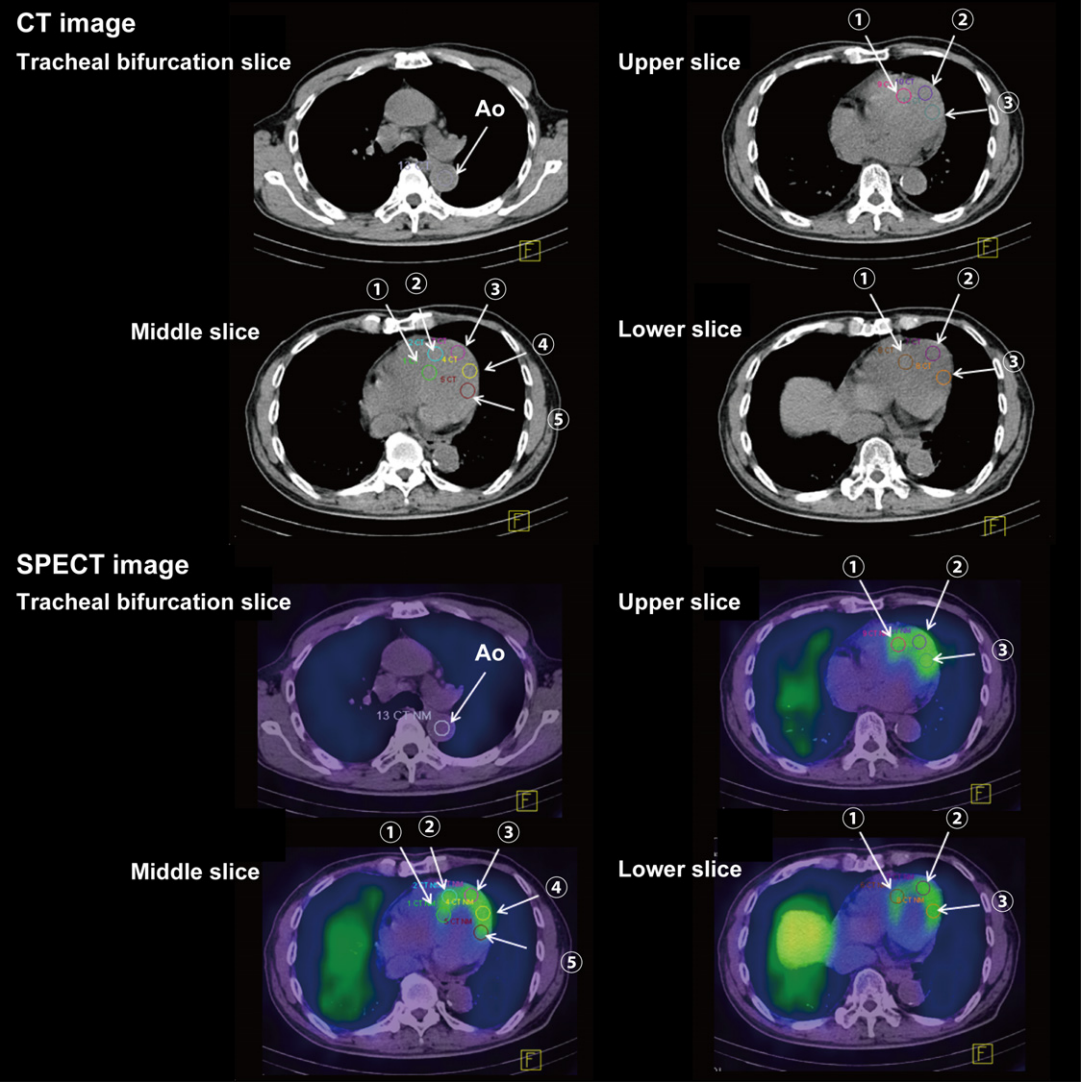
An example of manually defined ROIs on CT images and anatomical localization of ROIs on SPECT/CT fusion images. First, myocardial ROIs and an aorta ROI were placed on the CT images. Next, anatomical localization of.ROIs on a SPECT/CT fusion images were checked by the operator before the analysis.

For MIBG SPECT/CT images, we first selected 3 transverse heart slices from the obtained CT images (i.e., the middle, upper quartile and lower quartile slices). Next, we set 11 circular ROIs (15-mm diameters) on the left ventricular wall, 5 on the middle slice and 3 on each of the upper and lower quartile slices and then calculated the average counts. Next, we placed a 15-mm diameter circular ROI on the descending aorta at the level of the tracheal bifurcation. We then calculated the manual heart-to-aorta ratio (manual H/A ratio) in a manner similar to the planar method (Fig 1).

### Semi-automatic ROI measurement on SPECT/CT

Furthermore, we developed a semi-automatic MIBG SPECT/CT method. For this measurement, we extracted the heart boundary using in-house software written in Interactive Data Language (Exelis Visual Information Solutions, CO, USA), which was loaded on the workstation. We first selected 3 transverse heart slices in the manner described above. Second, we extracted the heart boundary from 3 CT slices based on the difference in absorption rates using above-mentioned in-house software. In this step, the operator firstly put a rough ROI encircling the heart manually. Second, the operator entered a density value that was intermediate relative to the values of the lungs and myocardium. Finally, the above-mentioned software automatically delineated the heart boundary and created 3 whole-heart ROIs on the CT images. We then applied these ROIs to the MIBG SPECT image and calculated the average count for each patient. Finally, we calculated the semi-automatatic heart-to-aorta ratio (semi-automatic H/A ratio) following the same process as the manual SPECT/CT method.

### Data Analysis

One-way analysis of variance (ANOVA) with post-hoc Tukey tests was used to compare the PD, DLB, and iNPH groups. The results are expressed as the mean±SD. P<0.05 was considered statistically significant. To compare the usefulness of these 3 methods of evaluation for discriminating between LBDs and iNPH, we performed a receiver operating characteristic (ROC) analysis. The difference in the area under the ROC curve measures among 3 methods was analyzed using chi-square test. Furthermore, we calculated the intraclass correlation coefficients (ICCs) to evaluate the interobserver reliabilities of the conventional planar ROI method and the manual and semi-automatic SPECT/CT methods. In this analysis, 3 radiology technologists blinded to the diagnosis independently calculated the conventional H/M ratios and the manual and semi-automatic H/A ratios of 18 patients who were randomly sampled from the total 54 patients. Next, we determined the ICCs of the 3 methods. The ICC analyses were performed using SPSS statistical software (version 21, SPSS Inc., Chicago, IL), and all other statistical analyses were performed using JMP 10.0.2 software (SAS Institute Inc., Cary, NC, USA).

## Results

**Fig 2:**
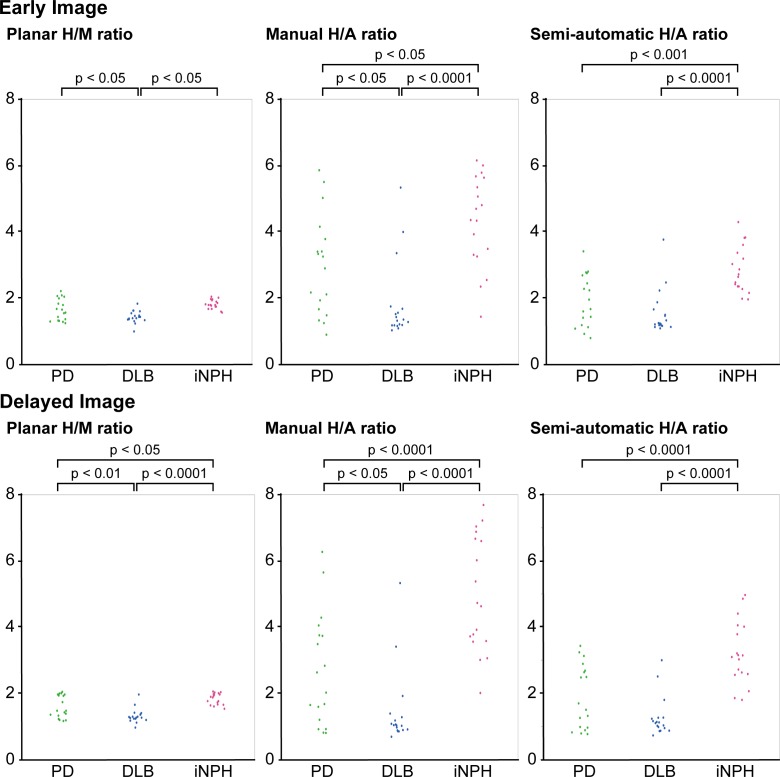
Comparison between the planar and SPECT/CT methods for estimation of cardiac ^123^I-MIBG uptake. The manual and semi-automatic SPECT/CT methods identified decreased cardiac uptake of ^123^I-MIBG in PD and DLB compared with iNPH in the early and delayed images. However, in the planar method, there were no significant differences in cardiac ^123^I-MIBG uptake in the early image.

The results of the ^123^I-MIBG scintigraphy are presented in Fig 2 and S1 Table. The planar H/M ratios in the early and delayed images were 1.64±0.33 and 1.59±0.34 for the PD group, 1.42±0.18 and 1.31±0.21 for the DLB group, and 1.80±0.14 and 1.83±0.17 for the iNPH group, the manual H/A ratios in the early and delayed images were 2.95±1.50 and 2.67±1.68 for the PD group, 1.79±1.12 and 1.42±1.15 for the DLB group, and 4.33±1.38 and 4.96±1.74 for the iNPH group, and the semi-automatic H/A ratios in the early and delayed images were 1.91±0.78 and 1.88±0.97 for the PD group, 1.55±0.68 and 1.26±0.59 for the DLB group, and 2.84±0.70 and 3.21±0.96 for the iNPH group, respectively. In the conventional planar ROI analysis, the DLB group exhibited significantly lower H/M ratios in both the early and delayed images compared with the PD and iNPH groups. The PD group exhibited a non-significant trend toward lower H/M ratios in the early images compared with the iNPH group. In contrast, the PD and DLB groups exhibited significantly lower cardiac uptakes of ^123^I-MIBG in both the early and delayed images in the manual and semi-automatic SPECT/CT analyses. The DLB group exhibited a non-significant trend toward lower H/A ratios in the early and delayed images compared with the PD group in the semi-automatic SPECT/CT analysis.

**Fig 3:**
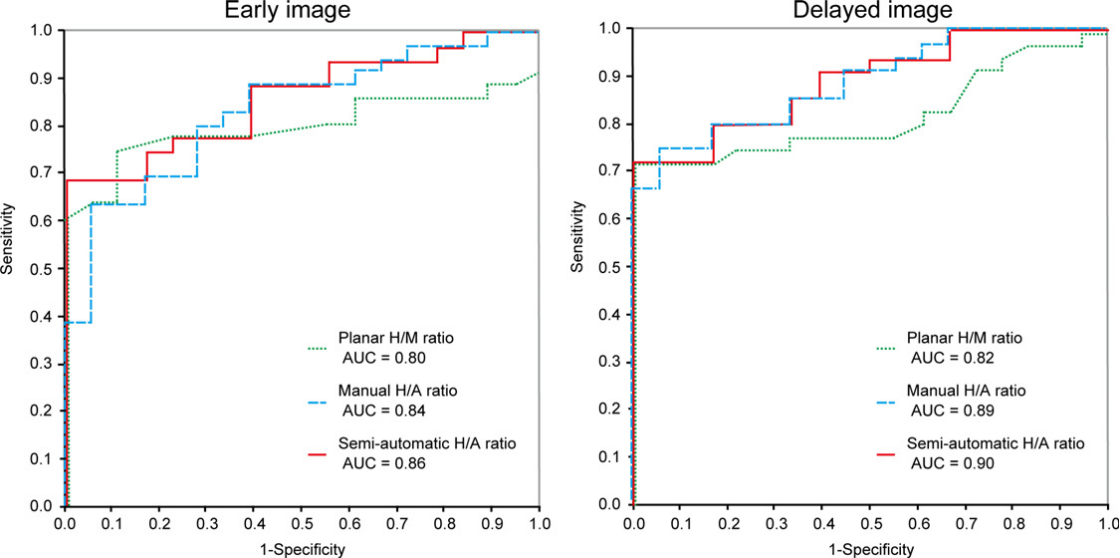
ROC curves for discriminating between the LBDs and iNPH groups. The measurements of both the manual and semi-automatic SPECT/CT methods, especially in the delayed image, had higher discriminative power compared to the planar method in diagnosing LBDs. ROC, receiver operating characteristic; LBDs, Lewy body diseases; AUC, area under the curve; H/M ratio, heart-to-mediastinum ratio; H/A ratio, heart-to-aorta ratio.

The results of the ROC analysis are presented in Fig 3. Both the manual and semi-automatic SPECT/CT methods produced higher AUCs than the conventional planar method, and statistically significant differences were observed in the delayed images. At the optimal cutoff values for differentiation, the respective sensitivities and specificities of the 3 methods for the early images were 75.0% and 88.9% for the conventional planar method, 63.9% and 94.4% for the manual SPECT/CT method, and 69.0% and 100% for the semi-automatic SPECT/CT method. Additionally, for the delayed images, the respective sensitivities and specificities of the 3 methods were 72.2% and 100% for the conventional planar method, 75.0% and 94.4% for the manual SPECT/CT method, and 72.0% and 100% for the semi-automatic SPECT/CT method.

The respective intraclass correlation coefficients were 0.987 and 0.996 for the early and delayed H/M ratios in the conventional planar method, 0.968 and 0.976 for the early and delayed H/A ratios in the manual SPECT/CT method, and 0.968 and 0.985 for the early and delayed H/A ratios in the semi-automatic SPECT/CT method.

## Discussion

The early detection of cardiac sympathetic dysfunction has a significant influence on the diagnosis and clinical management of neurodegenerative dementias [17]. Accumulating evidence suggests that ^123^I-MIBG cardiac scintigraphy is useful for evaluating cardiac sympathetic damage and improves diagnostic accuracy for LBDs [3, 5, 6, 18]. However, the sensitivity of MIBG scintigraphy remains low, particularly in the early stages of the disease [8, 9], and substantial room for improvement still exists. Increasing the accuracy of image analysis is, not surprisingly, a simple and effective solution to this problem. Based on this knowledge, we applied a SPECT/CT hybrid imaging technique to ^123^I-MIBG cardiac scintigraphy.

In this study, we compared the diagnostic utility of ^123^I-MIBG cardiac SPECT/CT imaging with that of conventional planar imaging for differentiating PD, DLB and iNPH, because these diseases sometimes share symptoms and we need specific tools to discriminate between LBDs and iNPH. In agreement with previous studies, the patients with LBDs exhibited reduced uptake of ^123^I-MIBG on both conventional planar scintigraphy and SPECT/CT hybrid imaging [5, 6]. As expected, the manual and semi-automatic SPECT/CT methods exhibited superior diagnostic performances, with satisfactorily high AUCs compared with the planar method. One possible reason for the advantage of the SPECT/CT method is that the broad dynamic range of the H/A ratio enabled a precise evaluation of cardiac dysfunction in PD, DLB and iNPH. In the present study, the dynamic range of the H/M ratio in the planar ROI method was 0.96–2.2. In contrast, the dynamic ranges of the H/A ratios were 0.68–7.68 for the manual SPECT/CT method and 0.72–4.96 for the semi-automatic SPECT/CT method. These results indicate that our SPECT/CT method exhibited a broad dynamic range of H/A ratios that was at least twice that of the conventional method. Therefore, the use of the hybrid SPECT/CT system could likely increase the dynamic range of the H/A ratio and aid in resolving equivocal cardiac scintigraphic findings. To our knowledge, these results provide the first evidence demonstrating that ^123^I-MIBG SPECT/CT imaging has a greater diagnostic utility for differentiating LBDs than the conventional planar method.

Furthermore, our manual and semi-automatic SPECT/CT methods produced additional advantages, including ease of use and exhibiting a high inter-rater reliability. Although the semi-automatic SPECT/CT method has a broader dynamic range, this method exhibited sufficiently high ICCs in the present study. The cardiac margins were easily determined, and the descending artery at the level of the tracheal bifurcation was readily identified on CT scans. For these reasons, the manual and semi-automatic SPECT/CT methods achieved consistent results and enabled a highly accurate evaluation of cardiac sympathetic dysfunction. In particular, the semi-automatic SPECT/CT method was free from observer bias, and thus expected to become a standard technique.

Our SPECT/CT ROI method may contribute to the early diagnosis and treatment of LBDs. Several recent imaging studies have demonstrated that abnormal ^123^I-MIBG cardiac scans preceded cognitive deterioration in LBDs [19]. Oda et al. demonstrated that abnormal ^123^I-MIBG cardiac scans heralded the conversion from mild dementia to DLB [20]. Furthermore, a recent study reported an association between low MIBG uptake and faster disease progression in PD [21]. Our method may offer better analytical performance in terms of ^123^I-MIBG abnormalities than the conventional method that was used in these studies. Therefore, ^123^I-MIBG SPECT/CT imaging could contribute to the prediction and early diagnosis of LBDs. However, further study is needed to confirm this hypothesis.

The limitations of this study include the relatively small sample size and the lack of pathological confirmation of PD and DLB. Furthermore, the utility of our SPECT/CT ROI method in the differential diagnosis of common parkinsonian disorders other than iNPH, such as vascular parkinsonism, progressive supranuclear palsy and multiple system atrophy, still be unproven. These limitations will be addressed in future research. However, the clinical diagnoses of PD and DLB were based on internationally accepted criteria, and the diagnosis of iNPH was based on the results of surgical treatment. Furthermore, SPECT/CT MIBG cardiac scintigraphy may have diagnostic utility, particularly in the early stages of the LBDs. Therefore, these limitations could exert insignificant effects on the value of this study.

In conclusion, we found that ^123^I-MIBG cardiac SPECT/CT imaging displayed superior diagnostic utility for cardiac sympathetic dysfunction in parkinsonian disorders. Furthermore, this SPECT/CT method had sufficiently high reliability. Our results provide a convenient tool for diagnosing LBDs in the early stages and allow for early intervention.

## Supporting Information

S1 Table The original data of demographics and the results of the ^123^I-MIBG scintigraphy in this study. (XLSX)
